# Identification of circulating microvesicle‐encapsulated miR‐223 as a potential novel biomarker for ARDS

**DOI:** 10.14814/phy2.15494

**Published:** 2022-11-10

**Authors:** Sultan Almuntashiri, Yohan Han, Hannah A. Youngblood, Aaron Chase, Yin Zhu, Xiaoyun Wang, Daniel F. Linder, Budder Siddiqui, Andrea Sikora, Yutao Liu, Duo Zhang

**Affiliations:** ^1^ Clinical and Experimental Therapeutics, College of Pharmacy University of Georgia and Charlie Norwood VA Medical Center Augusta Georgia USA; ^2^ Department of Clinical Pharmacy, College of Pharmacy University of Hail Hail Saudi Arabia; ^3^ Department of Cellular Biology and Anatomy Augusta University Augusta Georgia USA; ^4^ Department of Clinical and Administrative Pharmacy, College of Pharmacy University of Georgia Augusta Georgia USA; ^5^ Department of Pharmacy Augusta University Medical Center Augusta Georgia USA; ^6^ Division of Biostatistics and Data Science, Medical College of Georgia Augusta University Augusta Georgia USA; ^7^ Division of Infectious Diseases, Medical College of Georgia Augusta University Augusta Georgia USA; ^8^ Department of Medicine Augusta University Augusta Georgia USA

**Keywords:** acute lung injury, extracellular vesicles, inflammation, microRNA, neutrophil, sepsis

## Abstract

Acute respiratory distress syndrome (ARDS) is a lethal disease with severe forms conferring a mortality rate approaching 40%. The initial phase of ARDS results in acute lung injury (ALI) characterized by a severe inflammatory response and exudative alveolar flooding due to pulmonary capillary leak. Timely therapies to reduce ARDS mortality are limited by the lack of laboratory‐guided diagnostic biomarkers for ARDS. The purpose of this study was to evaluate the prognostic role of circulating microvesicles (MVs)‐containing miR‐223 (MV‐miR‐223) if indicate more severe lung injury and worse outcomes in ARDS patients. Human plasma samples from one hundred ARDS patients enrolled in Albuterol to Treat Acute Lung Injury (ALTA) trial were compared to a control group of twenty normal human plasma specimens. The amount of MV‐miR‐223 was measured using absolute real‐time polymerase chain reaction (PCR) with a standard curve. Mann–Whitney‐Wilcoxon, Spearman correlation, Chi‐squared tests, and Kaplan–Meier curves were computed to assess different variables and survival. Plasma levels of MV‐miR‐223 were significantly higher in ARDS patients compared to normal control subjects. Upon receiver operator characteristic (ROC) analysis of MV‐miR‐223 in relation to 30‐day mortality, MV‐miR‐223 had an area under the curve (AUC) of 0.7021 with an optimal cut‐off value of 2.413 pg/ml. Patients with high MV‐miR‐223 had higher 30‐day mortality than subjects with low MV‐miR‐223 levels. MV‐miR‐223 was negatively correlated with ICU‐free days, ventilator‐free days, and organ failure‐free days. Patients with high MV‐miR‐223 levels had higher 30 and 90‐day mortality. MV‐miR‐223 was associated with 28‐day clinical outcomes of ALTA trial including ICU‐free days, ventilator‐free days, and organ failure‐free days. Thus, circulating MV‐miR‐223 may be a potential biomarker in prognosticating patient‐centered outcomes and predicting mortality in ARDS.

## 1 INTRODUCTION

Acute respiratory distress syndrome (ARDS) is life‐threatening to critically ill patients without early intervention, but laboratory‐based tests for early diagnosis are lacking (Ragaller & Richter, 2010). The acute phase of ARDS and the associated acute lung injury (ALI) is characterized by exudative alveolar flooding due to pulmonary capillary leak and by extensive alveolar collapse due to loss of normal surfactant activity (Gonzales et al., 2015). This acute phase also marks the optimal time to initiate life‐saving interventions, but underdiagnosis rates up to 50% remain a key barrier to improved delivery of care (Parasher, 2021; Rezoagli et al., 2017). To date, no biomarkers have been confirmed for the diagnosis of ARDS or prediction of patient outcomes.

MicroRNAs (miRNAs) are a group of small non‐coding RNAs that regulate the expression of target genes at the post‐transcriptional level (O'Brien et al., 2018). Currently, accumulating data demonstrate miRNAs are key regulators that fine‐tune the expression of hundreds of target genes and are involved in a range of biological and human disease processes (Ardekani & Naeini, 2010; O'Brien et al., 2018). In particular, miR‐223 is a hematopoietic‐specific miRNA with crucial functions in myeloid lineage development and granulocytic differentiation whose deficiency has been associated with severe lung inflammation. Conversely, pulmonary overexpression of miR‐223 resulted in enhanced lung protection during mouse models of ALI induced by mechanical ventilation or by infection with *Staphylococcus aureus* (Fazi et al., 2005; Neudecker et al., 2017).

Microvesicles (MVs) are a form of extracellular vesicles (EVs). EVs are small membrane‐based vesicles secreted by almost all mammalian cells as cell‐to‐cell communication mediators in physiological and pathological scenarios (Yanez‐Mo et al., 2015). EVs carry a wide variety of DNA, RNA, proteins, and lipids and may serve as novel biomarkers in human diseases; however, limited evaluation of EVs in ARDS has been conducted (Yanez‐Mo et al., 2015). Our previous study has shown that circulating microvesicles (MVs) containing miR‐223 (MV‐miR‐223) were strikingly elevated during lipopolysaccharide (LPS)/live bacterial infection‐induced ALI in mouse models (Zhang et al., 2019). In this study, we hypothesized that high levels of circulating MV‐miR‐223 in ARDS patients would indicate more severe lung injury as measured by oxygenation status and worse patient‐centered outcomes.

## 2 MATERIALS AND METHODS

### 2.1 Study population

Plasma samples from 100 patients enrolled in Albuterol to Treat Acute Lung Injury (ALTA) trial were obtained through the Biologic Specimen and Data Repository Information Coordinating Center (BioLINCC) of the National Heart, Lung and Blood Institute (NHLBI). Plasma samples were collected from patients enrolled in the ALTA trial on the day of randomization. MV‐miR‐223 levels were retrospectively measured in our current study. The design of the ALTA trial has been described in detail previously (National Heart, Lung, and Blood Institute Acute Respiratory Distress Syndrome (ARDS) Clinical Trials Network et al., 2011). Briefly, 282 patients were enrolled and randomized to receive aerosolized albuterol or placebo to treat ALI between August 2007 and September 2008. Included patients had to have bilateral pulmonary infiltrates consistent with edema, partial pressure of oxygen to fraction of inspired oxygen (PaO_2_/FiO_2_ ratio) of 300 or less, and undergoing mechanical ventilation. Patients with clinical evidence of left atrial hypertension, chronic lung disease, chronic liver disease, neuromuscular disease, and acute myocardial infarction were excluded. For the control group, normal human plasma specimens (n = 20) were purchased from a commercial vendor (BIOIVT). MV‐miR‐223 levels from both ARDS and normal subjects were measured at the same time in our laboratory.

### 2.2 MV isolation and characterization

Human plasma specimens were filtered using a 0.8‐μm filter and MVs from 100 μl filtered plasma were prepared using sequential centrifugation protocols described previously (Zhang et al., 2019). Purified MVs (*n* = 4 per group) were examined using transmission electron microscopy (TEM) at Electron Microscopy Core Laboratory at Augusta University. The size of MVs (*n* = 3 per group) was determined using Nanoparticle Tracking Analysis (NTA) (ZetaView PMX 110, Particle Metrix).

### 2.3 Western blot analysis

The pooled plasma MVs from normal subjects (*n* = 5) and ARDS patients (*n* = 5) were characterized using Western blotting. MV‐specific marker proteins, ANXA1, ANXA2, and ITGB1 (Jeppesen et al., 2019), as well as a transcription factor SP1, which is not expressed in MVs (Zhang et al., 2017), were detected. THP‐1 cell lysate was used as a positive control group. Briefly, MVs and THP‐1 cells (ATCC, Manassas, VA) were lysed in RIPA buffer containing protease inhibitors. The protein concentration was measured using Pierce™ BCA (bicinchoninic acid) Protein Assay Kit (Thermo Fisher Scientific) according to the manufacturer's protocol. Proteins in lysed samples were separated on SDS‐PAGE gels and transferred to the PVDF membrane. Membranes were blocked in 5% BSA/TBST for 1 h at room temperature and incubated with primary antibodies with monoclonal anti‐ANXA1 (Abcam, ab214486), monoclonal anti‐ANXA2 (Abcam, ab178677), monoclonal anti‐ITGB1 (Abcam, ab52971), and polyclonal anti‐Sp1 (Abcam, ab227383) overnight at 4°C. The next day, membranes were incubated with polyclonal anti‐rabbit HRP secondary antibody (R&D Systems, HAF008) for 1 h. Images were captured by the Chemidoc image system (BioRad).

### 2.4 RNA isolation, miRNA reverse transcription, and absolute quantitative real‐time PCR

The amount of MV‐miR‐223, MV‐miR‐142, and MV‐miR‐182 in 100 μl plasma was measured from ARDS patients and the control group using absolute real‐time PCR with a standard curve (Zhang et al., 2018). MVs pelleted from 100 μl filtered plasma were subject to total RNA extraction using miRNeasy Mini Kit (Qiagen, Hilden, Germany). To measure the miR‐223, miR‐142, and miR‐182 expression, stem‐loop RT‐qPCR was performed as previously reported (Chen et al., 2005). Briefly, extracted RNA from each sample was used for generating the cDNA (high‐capacity cDNA reverse transcription kit, Applied Biosystems) for miRNA detection in a final volume of 10 μl; including 1 μl 10× RT Buffer, 0.4 μl 25× dNTP Mix, 0.4 μl Reverse Transcriptase, 0.25 μl 10 μM U6 RT primer and 0.25 μl 10 μM miRNA RT primer. The following primers were used: miR‐223 reverse transcription: 5′‐CTC AACTGGTGTCGTGGAGTCGGCAATTCAGTTGAGTGGGGTAT‐3′; miR‐223 qPCR forward: 5′‐ACACTCCAGCTGGGTGTCAGTTTGTCAAAT‐3′; qPCR universal reverse: 5′‐GGTGTCGTGGAGTCGGCAATTCAGTTGAG‐3′. miR‐142 reverse transcription: 5′‐ CTCAACTGGTGTCGTGGAGTCGGCAATTCAGTTGAGTCCATAAA‐3′; miR‐142 qPCR forward: 5′‐ ACACTCCAGCTGGGTGTAGTGTTTCCTACT‐3′; miR‐182 reverse transcription: 5′‐CTCAACTGGTGTCGTGGAGTCGGCAATTCAGTTGAGCGGTGTGA‐3′; miR‐182 qPCR forward: 5′‐ACACTCCAGCTGGGTTTGGCAATGGTAGAA‐3′. Real‐time RT‐PCR was performed by using the Ultra SYBR Two‐Step RT‐qPCR Kit (Thermo Fisher Scientific) according to the manufacturer's protocol.

### 2.5 Copy number of miR‐223 calculation

MiR‐223 copies per single MV were calculated as described previously (Chevillet et al., 2014). Briefly, the absolute miR‐223 copies in 100 μl plasma were divided by MV number from 100 μl plasma.

### 2.6 Statistical analysis

All statistical analyses were performed using IBM SPSS Statistics Version 27.0. GraphPad Prism was used to develop figures. The significance level was set at α = 0.05. Demographic data were evaluated using descriptive statistics. Differences in continuous variables among groups were assessed using the Mann–Whitney‐Wilcoxon (MWW) nonparametric test. The Spearman correlation coefficient was used to assess bivariate relationships between continuous variables. Chi‐squared tests were used to assess relationships between categorical variables. An area under the receiver operating characteristic (AUROC) analysis was performed to assess the predictive power of MV‐miR‐223 for 30‐day mortality, and the optimal cutoff level of MV‐miR‐223 was determined using Youden's index. Finally, Kaplan–Meier survival curves were computed for the MV‐miR‐223 groups that were determined by the AUROC analysis, and a log‐rank test was used to determine survival differences between these groups.

## 3 RESULTS

### 3.1 Study population

The demographic information of 20 normal control subjects and 100 ARDS patients enrolled in the ALTA trial is shown in Table 1. Significant differences are found between these two groups in age, sex, and race.

**TABLE 1:** Demographic and summary characteristics of human subjects

Characteristic	Normal control (*n* = 20)	ARDS (*n* = 100)	*p* value
Age y (*SD*)	39.25 (10.467)	50.76 (15.561)	0.002
Female sex *n* (%)	15 (75)	46 (46)	0.018
Race			
Black *n* (%)	5 (25)	18 (18)	0.001
White *n* (%)	0 (0)	72 (72)	
Hispanic *n* (%)	15 (75)	4 (4)	
Other *n* (%)	0 (0)	6 (6)	
Primary cause of ARDS, *n* (%)			
Aspiration	—	16 (16)	—
Multiple transfusion	—	2 (2)	
Pneumonia	—	38 (38)	
Sepsis	—	28 (28)	
Trauma	—	10 (10)	
Other	—	6 (6)	

### 3.2 Purification and characterization of MVs derived from human plasma

**FIGURE 1:**
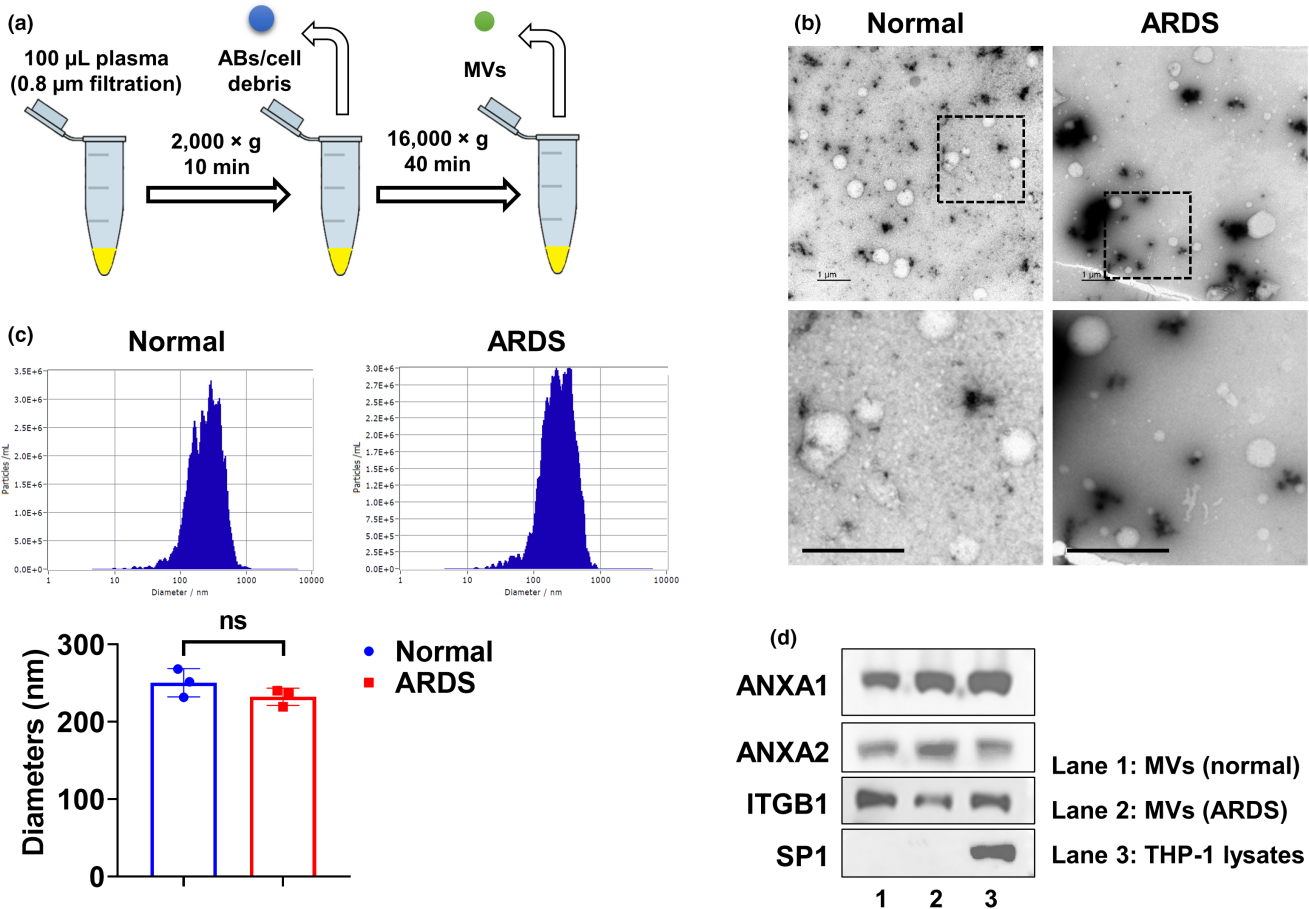
Purification and characterization of MVs derived from human plasma. (a) Schematic illustration of MV purification using sequential centrifugation protocols. (b) TEM images of plasma MVs (*n* = 4 per group) were shown, scale bar = 1 μm. (c) The diameters of isolated MVs (*n* = 3 per group) were measured using NTA. (d) MV positive markers (ANXA1, ANXA2, and ITGB1) and a negative marker (SP1) were detected in 100 μg pooled MV protein from the normal control group (*n* = 5), ARDS group (*n* = 5), and 20 μg THP‐1 cell lysate using western blot. ns, *p* > 0.05.

MVs were purified from control and ARDS groups using sequential centrifugation protocols (Figure 1a). Both MVs from different groups have a typical round shape (Figure 1b). Moreover, there was no difference in the diameters of isolated MVs between groups (Figure 1c). As shown in Figure 1d, MV marker proteins, ANXA1, ANXA2, and ITGB1, were detected in lysates of MVs and THP‐1 cells. Sp1 transcription factor was only detected in cell lysate confirming that plasma MVs were successfully purified. These results indicate that circulating MVs can be detected and purified from plasma samples of normal control subjects and ARDS patients.

### 3.3 Plasma MV‐miR‐223 levels in normal control subjects and ARDS patients

**FIGURE 2:**
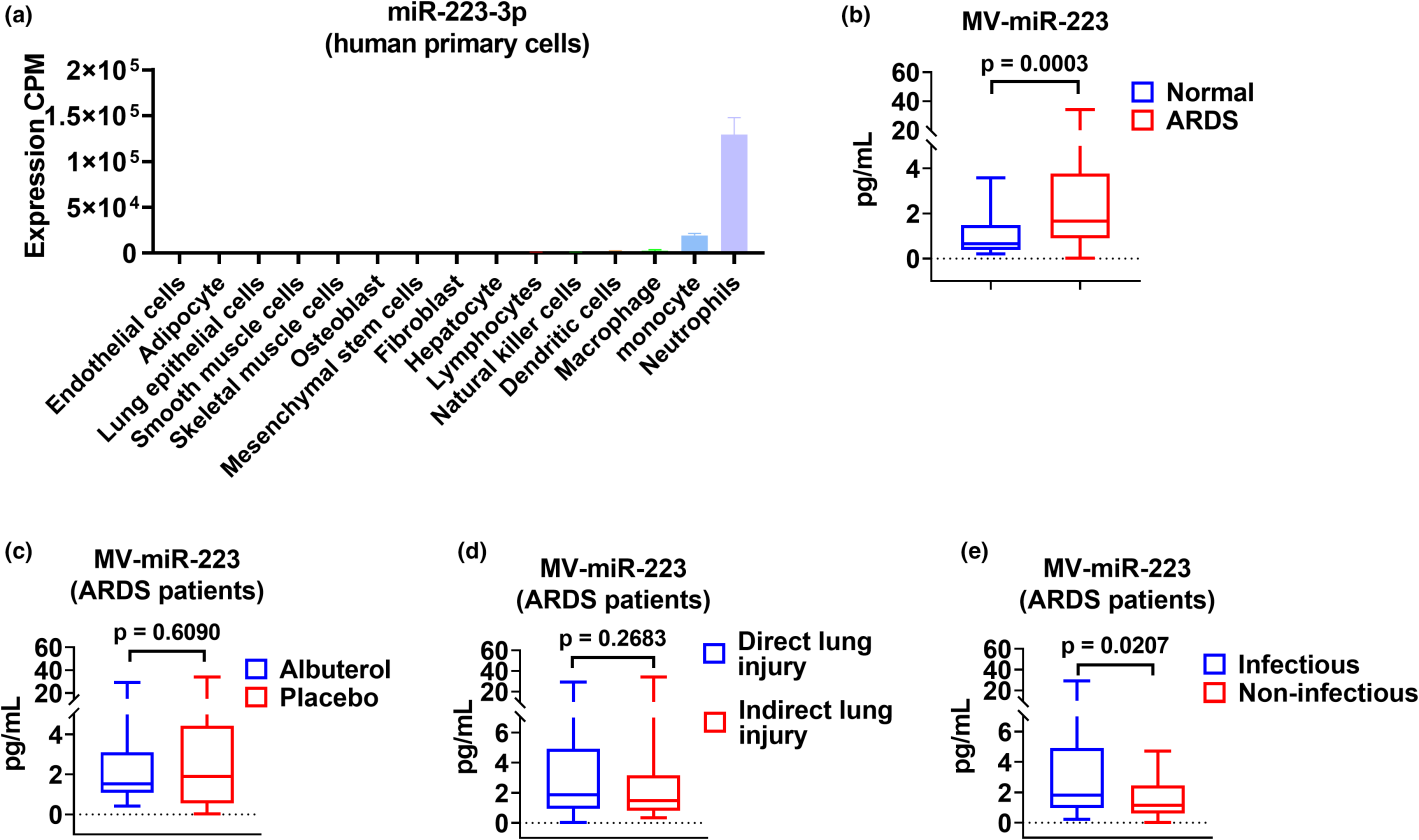
Comparison of plasma MV‐miR‐223 in different groups. (a) miR‐223 expression profile in various human primary cells from FANTOM5 database accessed on 05/18/2022. (b) normal control (*n* = 20) vs ARDS (*n* = 100). (c) Albuterol (*n* = 50) vs placebo (*n* = 50). (d) Direct lung injury (*n* = 54) vs indirect lung injury (*n* = 46) and (e) infectious (*n* = 66) vs non‐infectious (*n* = 28).

*miR‐223* is highly conserved in mammals and located on the X chromosome. It has been reported that miR‐223 is a hematopoietic‐specific miRNA (Fazi et al., 2005). The expression profile from the FANTOM5 database (accessed 05/18/2022) also supports the conclusion. Notably, miR‐223 is highly enriched in neutrophils (Figure 2a). Next, MVs were purified from control and ARDS groups using sequential centrifugation protocols. The amount of plasma MV‐miR‐223 from ARDS patients was compared to the normal control group. Plasma levels of MV‐miR‐223 were significantly higher in ARDS patients when compared with levels in normal control subjects. The median level of MV‐miR‐223 in the control group (*n* = 20) was 0.655 pg/ml. In contrast, the median level of MV‐miR‐223 from ARDS patients (*n* = 100) was 1.649 pg/ml (Figure 2b, *p* = 0.0003 ARDS vs. normal). These results indicate that more miR‐223 is secreted via MV in ARDS patients when compared to those from normal controls.

### 3.4 Plasma MV‐miR‐223 levels in various subgroups of ARDS patients

The ALTA trial aimed to evaluate the efficacy of albuterol compared to placebo in ALI. The effects of these two treatments on MV‐miR‐223 concentration were evaluated with no significant difference observed between albuterol and placebo groups (Figure 2c, *p* = 0.6090 albuterol vs placebo). Next, direct (aspiration and pneumonia) versus indirect causes (sepsis, trauma, and multiple transfusion) of ARDS were evaluated with no significant difference observed (Figure 2d, *p* = 0.2683 direct vs indirect lung injury). Interestingly, ARDS patients with infectious etiologies (sepsis and pneumonia) had significantly higher MV‐miR‐223 when compared to those with non‐infectious etiologies (aspiration, multiple transfusion, and trauma). (Figure 2e, infectious vs non‐infectious 1.809 pg/ml vs. 1.149 pg/ml, *p* = 0.0207). These findings indicate the involvement of miR‐223 in the inflammatory process.

### 3.5 Plasma MV‐miR‐223 levels and mortality

**FIGURE 3:**
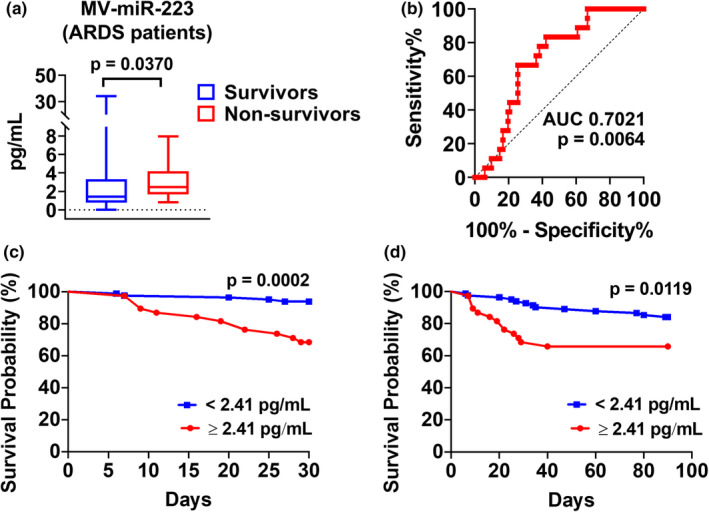
Plasma MV‐miR‐223 levels and mortality. (a) Survivors (*n* = 82) vs. non‐survivors (*n* = 18) at day 30. (b) MV‐miR‐223 distinguishes survivors from non‐survivors (area under the receiver operating characteristic curve [AUC] = 0.7021). A cut‐off of 2.413 pg/ml provided the highest AUC. (c) Kaplan–Meier survival curves for ARDS patients censored at 30‐days follow‐up. (d) Kaplan–Meier survival curves for ARDS patients censored at 90‐days follow‐up.

To assess the MV‐miR‐223 in relation to 30‐day mortality, plasma MV‐miR‐223 levels from survivors and non‐survivors were measured. Subjects with 30‐day mortality had significantly higher plasma MV‐miR‐223 when compared to survivors. (Figure 3a, survivors vs. non‐survivors at day 30, 1.431 pg/ml vs. 2.478 pg/ml, *p* = 0.0370). Upon AUROC analysis of miR‐223 in relation to 30‐day mortality, MV‐miR‐223 had an AUC of 0.7021 (Figure 3b, 95% confidence interval [CI]: 0.5956 to 0.8085; *p =* 0.0064) with an optimal cut off value of 2.413 pg/ml producing a 66.67% sensitivity and 74.51% specificity. When subjects were grouped as high (≥2.413 pg/ml) or low (<2.413 pg/ml) miR‐223 levels, the high miR‐223 group had an increased 30‐day mortality rate (31.58 vs 6.1%, *p* = 0.0002) and significantly increased hazards of mortality using time‐to‐event analysis censored at 30‐days follow‐up as compared to the low miR‐223 group (Figure 3c). When stratified by the same cut‐off level, the high miR‐223 group also had a higher 90‐day mortality rate but with a less significant difference between the groups (34.21 vs 15.86%, *p* = 0.0119; Figure 3d). These results indicate that circulating MV‐miR‐223 might serve as a biomarker for the early prediction of mortality.

### 3.6 MV‐miR‐223 and its level correlates with the outcomes of ARDS patients

**FIGURE 4:**
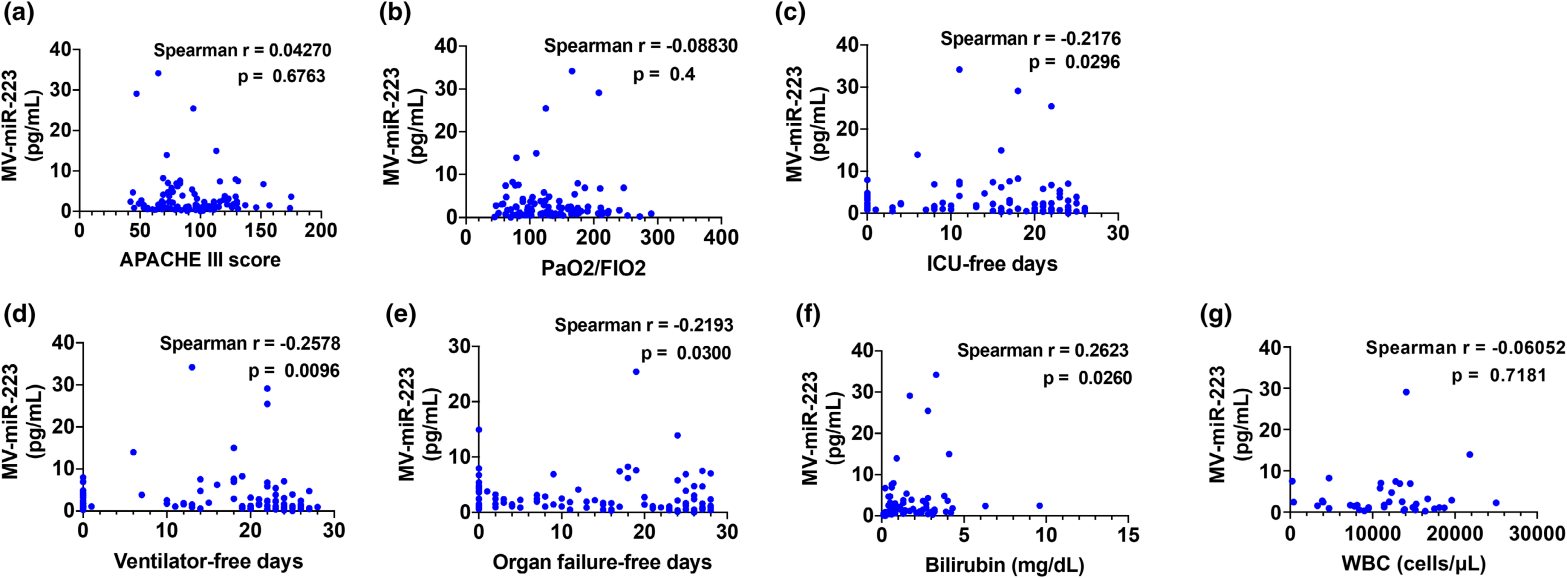
Correlation analyses between plasma MV‐miR‐223 and the prognostic parameters in ARDS patients. Spearman correlation coefficient was used to test relationships between (a) MV‐miR‐223 and APACHE III score (*n* = 98), (b) MV‐miR‐223 and PaO_2_/FIO_2_ ratio (*n* = 93), (c) MV‐miR‐223 and ICU‐free days (*n* = 100), (d) MV‐miR‐223 and ventilator‐free days (*n* = 100), (e) MV‐miR‐223 and organs failure‐free days (*n* = 98), (f) MV‐miR‐223 and bilirubin (*n* = 72). (g) MV‐miR‐223 and WBC counts (*n* = 38).

When the correlation of MV‐miR‐223 with prognostic parameters was assessed, there were no significant correlations between MV‐miR‐223 level and APACHE III score or PaO_2_/FiO_2_ ratio (Figure 4a,b). When assessing correlations between MV‐miR‐223 level and patient‐specific outcomes, a significant negative correlation was observed between ICU‐free days at 28 days and plasma MV‐miR‐223 levels in ARDS patients (Figure 4c, *r*  = − 0.2176, *p* = 0.0296). Similarly, there was a significant negative correlation between plasma MV‐miR‐223 levels and ventilator‐free days (Figure 4d, *r*  = − 0.2578, *p* = 0.0096) and organ failure‐free days (Figure 4e, *r* = − 0.2193, *p* = 0.03). These data suggest that plasma MV‐miR‐223 may reflect the clinical severity of ARDS. Dysregulation of miR‐223 had been previously reported in several liver diseases including cirrhosis, viral hepatitis, alcohol‐induced lung injury and hepatocellular carcinoma (Ye et al., 2018). We further analyzed the correlation between MV‐miR‐223 and bilirubin levels and found they were positively correlated, which indicates MV‐miR‐223 may reflect liver injury in ARDS patients (Figure 4f, *r* = 0.2623, *p* = 0.0260). Given that miR‐223 is highly enriched in neutrophils, we did a correlation analysis between MV‐miR‐223 levels and WBCs but no significant difference was noticed. (Figure 4g, *r* = −0.0605, *p* = 0.7181).

### 3.7 The copies of miR‐223 per MV but not MV numbers are increased in plasma from ARDS patients

**FIGURE 5:**
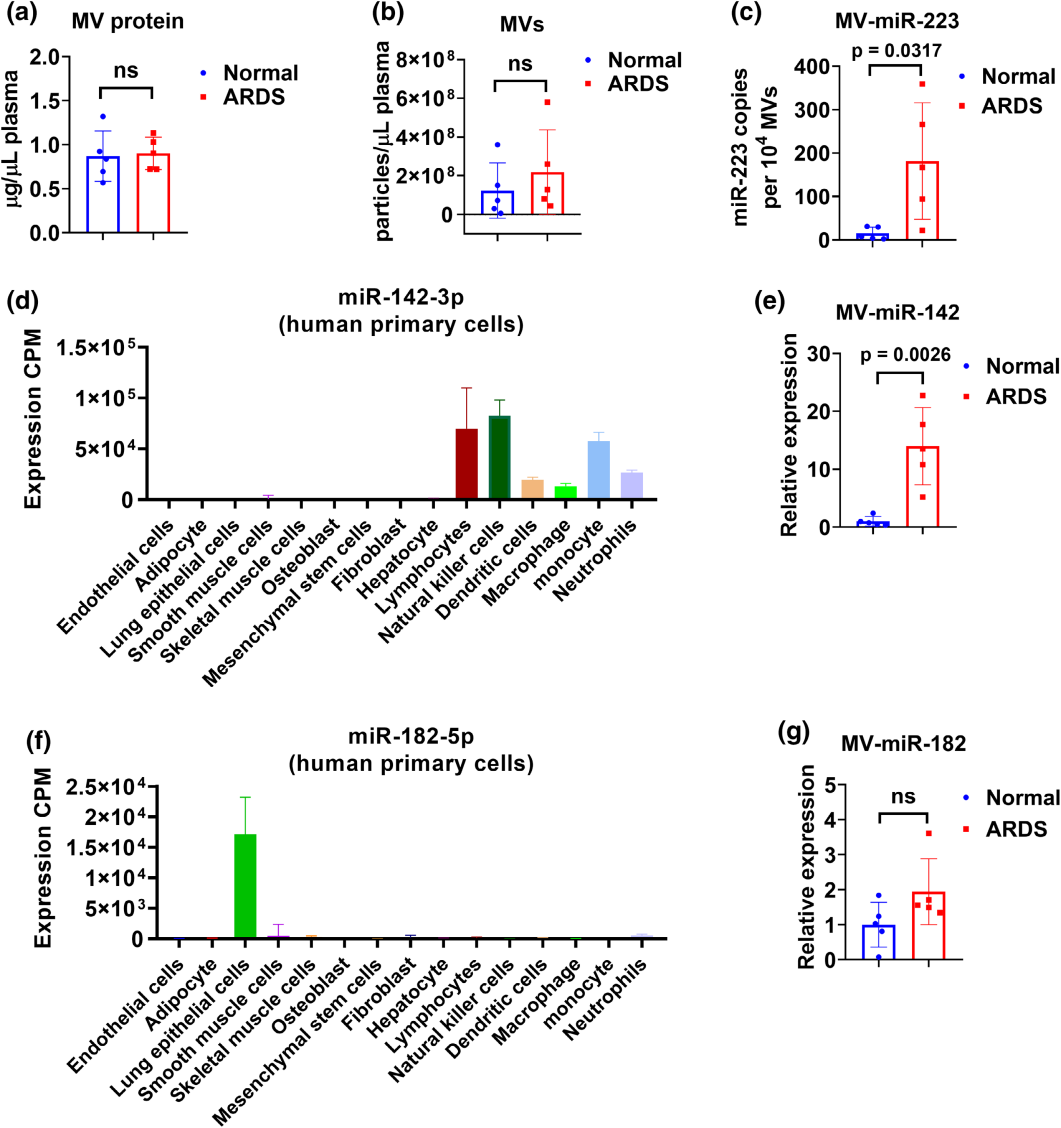
The copies of miR‐223 per MV are increased in ARDS patients. Plasma MVs were isolated from normal subjects and ARDS patients (*n* = 5 per group) using sequential centrifugation protocols. (a) The MV protein concentration is determined using BCA assay. (b) The number of MV particles is measured using NTA. (c) The copy number of miR‐223 in each MV was calculated. (d) miR‐142 expression profile in various human primary cells from FANTOM5 database accessed on 09/23/2022. (e) The relative levels of plasma MV‐miR‐142 in normal subjects and ARDS patients. (f) miR‐182 expression profile in various human primary cells from FANTOM5 database accessed on 09/23/2022. (g) The relative levels of plasma MV‐miR‐182 in normal subjects and ARDS patients. ns, *p* > 0.05.

As shown in Figure 1a, miR‐223 was measured in MV‐derived RNA isolated from 100 μl plasma. In this scenario, the elevated miR‐223 may come from: (1) a greater number of MVs in patients with ARDS; (2) increased copies of miR‐223 per MV; (3) both increased copies of miR‐223 and increased concentration of MVs. To address this question, we compared MV protein concentration using BCA protein assay and particle number per μl of plasma using NTA, respectively. However, we failed to see significant changes in protein amount or particle number between the normal and ARDS group (Figure 5a,b). Then, we calculated the miR‐223 copy number per MV and found that miR‐223 copies are significantly increased (Figure 5c). To be noted, even though miR‐223 is strikingly increased in MVs from the ARDS group, its copy number is still less than one copy per MV, which is in line with a previous study using serum exosomes (Chevillet et al., 2014). Furthermore, additional two miRNAs were detected to further confirm our observation. As shown in Figure 5d, miR‐142 is enriched in natural killer cells, lymphocytes, monocyte, neutrophils, dendritic cells, and macrophages (FANTOM5 database, accessed 09/23/2022). We confirmed that MV‐miR‐142 is also elevated in the ARDS group, which is consistent with our previous findings in mice treated with LPS and bacteria (Zhang et al., 2019; Figure 5e). In contrast, miR‐182 is highly expressed in lung epithelial cells (FANTOM5 database, accessed 09/23/2022) and it plays a role in pathways underlying tumor biology as a potential oncomir (Krishnan et al., 2013). Our data showed that MV‐miR‐182 has no significant change between these two groups (Figure 5g).

## 4 DISCUSSION

In our observational study, MV‐miR‐223 demonstrated diagnostic potential in differentiating ARDS compared to controls and prognostic potential for patient‐centered outcomes including 30 and 90‐day mortality, ICU‐free days, ventilator‐free days, and organ failure‐free days.

In the exudative stage of ARDS, inflammatory cells including neutrophils transmigrate into the lung leading to formation of hyaline membranes and irreversible damage to the lung characteristic of ARDS (Matthay et al., 2019). Markers of pro‐inflammatory monocyte–macrophage differentiation and neutrophil recruitment, such as miR‐223, are thus of interest for the diagnosis and prognosis of ARDS (Bauernfeind et al., 2012; Brook et al., 2019; Houshmandfar et al., 2021). Additionally, therapeutic targeting of miR‐223 may dampen excessive macrophage activation and neutrophil migration in inflammatory conditions (Yuan et al., 2018). Our previous study showed that serum MV‐containing miR‐223 is robustly secreted after bacterial‐induced ALI animal models (Zhang et al., 2019). Here, we continued the study and found evidence that MV‐miR‐223 is associated with clinical outcomes including mortality. We also found a significant difference in MV‐miR‐223 levels between infectious and non‐infectious subgroups. These results may indicate that a high level of plasma MV‐miR‐223 denotes more severe lung inflammation and worse outcomes in ARDS patients. In addition, miR‐223 itself could be a key regulator of the inflammatory reaction during lung injury, specifically to infection‐induced ALI.

Elevated levels of monocytes and neutrophils in circulation led to poor prognosis and early death in different diseases including interstitial lung disease (Saku et al., 2021) and melanoma (Schmidt et al., 2005). Similarly, both neutrophils and monocytes from sepsis patients were independent predictors of mortality on 28 days (Liu et al., 2021). MiR‐223 is abundant in circulating neutrophils and monocytes under normal physiology (Brook et al., 2019). Moreover, the life spans of both neutrophils and monocytes in circulation are very short and most likely not exceed a day (Lahoz‐Beneytez et al., 2016; Patel et al., 2017). However, miR‐223 released from such cells possibly remains in circulation for an extended period due to their long half‐life and relatively short sequences (Coenen‐Stass et al., 2019) suggesting a possibility of MV‐miR‐223 serves as a stable biomarker for ARDS. Although miR‐223 is highly enriched in neutrophils, we failed to see a significant correlation between MV‐miR‐223 levels and WBCs. This could be explained by the small sample size. In this study, only 38 ARDS patients have WBC count in the dataset and it was measured 24 h before randomization, which was not at the same time point as plasma was collected. Moreover, the data stand for WBCs, including not only neutrophils but also monocytes and lymphocytes. Therefore, it is worthy to further test the exact neutrophil or monocyte amounts in relation to MV‐miR‐223 levels in future studies.

In this study, we also showed that the increased copies of miR‐223 per MV contribute to the elevated MV‐miR‐223. We did not observe any significant changes in MV protein amount or particle numbers between the normal and ARDS group, indicating that the MV concentration in the circulation could be unchanged in the ARDS patients. Besides, the data from MV‐miR‐142 suggest that MV‐miR‐223 is not the only increased MV‐miRNA in ARDS patients. Also, not all the MV‐miRNAs are increased in ARDS patients as demonstrated by MV‐miR‐182.

On the other hand, the mechanism by which MV‐miR‐223 is released is largely unknown. Previously, we demonstrated that miRNA 3′ end uridylation mediates the packing of miR‐223 into MVs and the release of miR‐223 from macrophages via MVs mediates the inflammasome activation (Zhang et al., 2019). Nevertheless, further investigations are needed to determine whether circulating MV‐miR‐223 participates in the pathogenesis of ARDS.

Among several critical outcomes of ALTA trial, MV‐miR‐223 was negatively correlated with ICU‐free days, ventilator‐free days, and organ failure‐free days with higher levels of MV‐miR‐223 resulting in worse outcomes. While these indicate clinical severity of the disease, MV‐miR‐223 was not correlated with PaO_2_/FiO_2_ ratio or APACHE III score. This finding may be because oxygenation indices and APACHE III are not directly reflective of the severity of systemic inflammation and thus may offer novel insights to clinicians beyond traditional markers of disease severity.

Our study has several limitations. First of all, the control and ARDS plasma samples were not from the same trial. Therefore, the baseline characteristics of these subjects, including age, sex, and race are not balanced. Thus, a better control group, such as non‐ARDS in critically ill patients, is needed to validate the increase of circulating MV‐miR‐223 in ARDS patients. Moreover, we had a relatively small sample size from one cohort and this pilot study is retrospective in design, which makes the conclusion less convincing. Another challenge comes from the absolute quantification of miR‐223 using a standard curve. We found variation from 0.018 to 34.194 pg/ml in the ARDS group, which is probably caused by low accuracy and poor reproducibility using a standard curve. ddPCR has been applied to the quantification of circulating miRNAs and Hindson, etc. found ddPCR was superior to qPCR for quantifying circulating miRNAs (Hindson et al., 2013). In our future study, we plan to validate the results using droplet digital PCR (ddPCR).

In conclusion, plasma MV‐miR‐223 levels in a randomized, controlled trial of ARDS patients were associated with clinical outcomes including mortality, ICU‐free days, ventilator‐free days, and organ failure‐free days. Circulating MV‐miR‐223 may be a potential biomarker for inflammatory reaction, and mortality prediction in ARDS.

## AUTHOR CONTRIBUTIONS

DZ, SA and AS designed the research; SA, YH and HAY performed experiments; SA, YH, DFL, AC and YZ collected data, analyzed, and interpreted data. YH, XW, AC, BS, DFL, AS and YL discussed the results and reviewed the manuscript for important intellectual content. SA, YH, AC, AS and DZ wrote the manuscript. All authors read and approved the final manuscript.

## FUNDING INFORMATION

This work was supported by National Institutes of Health (NIH) grants NIH/NHLBI R00 HL141685 and NIH/NIAID R03 AI152003 to DZ. American College of Clinical Pharmacy Foundation Futures Grants Program to AC. NIH/NHLBI F32HL147437 and NIH/NIAID R03 AI169063 to XW.

## CONFLICT OF INTEREST

The authors declare that they have no competing interests. The funders had no role in the design of the study; in the collection, analyses, or interpretation of data; in the writing of the manuscript, or in the decision to publish the results.

## ETHICS STATEMENT

Public‐use data and plasma samples were collected from a previously conducted study, ALTA trial and provided via BioLINCC. All human procedures in ALTA trial were approved by the institutional review boards and written informed consents were obtained from patients or surrogates at each participating hospital. Plasma specimens were measured at Augusta University laboratories and this study was approved by the Augusta University Institutional Review Board (IRB number: 1128838–13) prior to its initiation.
